# How Does the Concept of *Guanxi-circle* Contribute to Community Building in Alternative Food Networks? Six Case Studies from China

**DOI:** 10.3390/bs12110432

**Published:** 2022-11-02

**Authors:** Yanyan Li, Zhenzhong Si, Yuxin Miao, Li Zhou

**Affiliations:** 1 School of Public Policy and Management, Tsinghua University, Beijing 100084, China; 2 Balsillie School of International Affairs, Waterloo, ON N2L 6C2, Canada; 3 Precision Agriculture Center, Department of Soil, Water and Climate, University of Minnesota, St. Paul, MN 55108, USA; 4 School of Agricultural Economics and Rural Development, Renmin University of China, Beijing 100872, China

**Keywords:** community building, cognitive trust, emotional trust, *guanxi*, *guanxi-circle*, alternative food networks

## Abstract

As social innovations that help to transition towards a more sustainable food system, alternative food networks (AFNs) in China have attracted much scholarly attention in recent years. However, studies of the community building behavior of AFNs at the micro-level in the Chinese social context are scant. Through in-depth case studies conducted between 2017 and 2021 and social network analysis, our study examines how founders of AFNs successfully facilitate community building among their customers. We find that in China, the traditional social-cultural construct, *guanxi*, plays a critical role in AFNs’ community formation and expansion. The study identifies a three-stage framework for understanding the community building process of AFNs. First, a group of *guanxi* of the same kind would form a *guanxi-circle*. Second, the initial *guanxi-circle* is enhanced and expanded to multiple secondary *guanxi-circles*. Third, these multiple *guanxi-circles* together and the interactions among them constitute the community of AFNs. We argue that to strengthen the community, AFNs operators should inspire key members to form secondary *guanxi-circles* by enhancing their cognitive trust and emotional trust.

## 1. Introduction

Conventional food systems have resulted in food insecurity and malnutrition, global resource constrains and intensive fossil-fuel dependence, interrelated ecological and livelihood crises, and the spread of non-communicable diseases associated with unhealthy diets and industrially processed foods [1,2]. Alternative food networks (AFNs) have emerged in response to these glaring challenges. Activists have mapped out different ways of addressing these challenges by creating new spaces for the production, consumption and trading of food, whose alternative ethical features (e.g., organic, fair trade, local, quality, slow) distinguish them from conventional products [3,4]).

In China, AFNs, including community supported agriculture (CSAs), farmers’ markets, and other food social enterprises have been warmly welcome by Chinese consumers because of their proved capacity of addressing public food safety anxiety by producing and supplying safe and high-quality food [5]. AFNs are also believed to have contributed to environmental sustainability through ecological food production, the experimentation of innovative agricultural practices and short food supply chains. They nourish and demonstrate the multi-functionalities of agriculture, calling upon the return of ecological values and social responsibilities to agriculture [6]. Many of the AFNs are social enterprises that are entrepreneurial initiatives with specific social and environmental goals, such as promoting fair trade and supporting new ecological farmers, rather than maximizing profit [7]. 

A decade after their introduction to China, the number of AFNs has grown rapidly to more than 2000 in 2022, fostered by heightened food safety concerns and a growing interest in sustainably and locally-produced food. However, their development has met bottlenecks in recent years, and they remain marginal in the agrifood system with small consumer base [8]. Therefore, they have not demonstrated the transformative impacts scholars claim on restructuring the conventional industrialized food system [9]. Global alternative food practices are also challenged by how to establish and maintain a trustful community [10]. Expanding and enhancing the “community” becomes a tough challenge that AFNs operators and food activists must address, not only for a more economically viable future of AFNs but also for the sustainable transition of China’s food system at large [11,12].

This paper aims to examine the community building process of Chinese AFNs. It first provides an overview of the key concepts involved in the study. It then explains the research methods used, including the case design, data collection and analytical approaches. Then, we focus on the process of community building and maintenance in six cases of AFNs and propose a three-stage framework to understand the community building process in AFNs. Based on these results, we make two recommendations for AFNs operators that will be useful for establishing and strengthening their community and thus contributing to the transition toward a more sustainable food system. 

## 2. Theory

### 2.1. Community Building Behavior

Existing studies on AFNs in China underscored drivers for their emergence and growth prosperity, the challenges they encounter in building consumer trust and in scaling up, adapted operational models, and the impacts they have on ecological agricultural sector, and their contradictory relationship with the conventional food supply chains [13,14,15,16]. It is widely recognized that food safety concerns [17] and the rising middle class have fueled the rapid growth of AFNs in China. However, when examining the conceptualization and theorization of “community” in AFNs, we found limited specific studies grounded in a close scrutiny of cases. 

Previous studies point out that “community” might be formed through place, shared interests, advocacy or profession, and community members who receive support from their communities and feel connected to others based on contact and commonalities [18,19]. Communication plays an important role in community building since emotional connections among community members are strengthened by frequent and positive communications [20]. As the community member feel satisfied through communication, their connection is strengthened and greater loyalty to the community is achieved; thus, community building will be more successful [21,22]. Recent studies have highlighted that online communication using social media, in particular, can enhance the sense of community through key variables such as trust.

AFNs’ community building strategies differ a lot when comparing cases in China with those in other countries. The initiators in China manage to build reflexive and inclusive practices and use social media to voice dissent and extend their reach [23]. In developed countries where AFNs emerged more than 40 years earlier than China, such as Canada, AFN founders belong to and are actively involved in various civil society organizations, and many take leadership roles in food movements that promote sustainable and just food systems [24]. They are able to expand the AFN movement and have a voice in shaping social, economic, and ecological policies that matter to them through these organizations and coalitions. They attract and unite community members by collectively starting the movements, such as anti-GMO campaigns, creation of local food system roundtables, advocacy for more scale-appropriate food regulations, and supporting international food sovereignty organizations such as La Via Campesina. In contrast, AFNs in China, despite their rapid development and national initiatives such as the annual CSA conference, are less involved in advocacy work that promotes wider social agendas or policy changes. The community building behavior this paper studied is not, therefore, among initiatives for food system transformations but among customers and between producers and their customers of specific initiatives for customer retention. 

### 2.2. Cognitive Trust and Emotional Trust

Scholars have shown that reconvening the trust between farmers and consumers is one of the key alternatives of AFNs [25]. Trust is a positive expectation from others in a risky situation (i.e., food safety risks in this case) [26]. The trust and communications within a group of people sharing same values, such as ethical consumption, fair trade, environmental protection, and supporting rural development, shape the community of AFNs [27]. In the process of AFNs’ community building, trust is established and maintained in various ways among multiple stakeholders. Trust is the core of community building among Chinese AFNs because of China’s notorious food safety scandals. It is well-known that the melamine milk scandal outbreak in 2008 severely damaged the credibility of Chinese-made goods. China Central Television’s survey conducted in 2011 found that 70% of Chinese people were afraid to buy domestic milk. This scandal followed by a series of other food safety scandals resulted in a food safety crisis in China that deteriorated people’s trust in food, making trust a high priority in AFNs’ community building [28,29]. The cultural distance between consumers and conventional food producers also creates a critical barrier for AFNs and ecological food producers [8].

McAllister [30] classified trust into two categories: cognitive trust and emotional trust. Cognitive trust refers to the cognitive judgment on the reliability and professional ability of the trusted party. Emotional trust is based on interpersonal relationships and focuses on personal interactions and experience. Cognitive trust is based on empirical evidence and rational thought processes and is complemented by emotional bonds between those who participate in the trust relationship [31,32]. Researchers have argued that cognitive trust could foster emotional trust and vice versa. This classification of trust has been verified by previous empirical studies on Chinese AFNs. For instance, Schumilas et al. [23] argued that people in the community of CSAs share a highly cognitive trust, while Chen [33] and Chen [34] found that consumers could attain high emotional values in CSAs. The classification between cognitive trust and emotional trust provides a framework for us to unpack the community building behavior in AFNs.

### 2.3. Guanxi and Guanxi-circle

Although AFNs constitute a crucial force to boost the growth of the ecological agricultural sector in China [5], they face critical barriers, including high food price, limited food variety, short delivery season and difficulty in scaling up [35]. To overcome these barriers, AFNs in China have compromised in various ways with the mainstream conventional food system despite their often-confrontational values. A key finding from previous studies is that the challenge of community building reflects one critical factor of AFNs—reconnecting producers and consumers. Oftentimes, the producers here in this trust relationship refer to the managers or founders of these AFNs, rather than the real farmers working on the farm. Although researchers acknowledge the significance of consumer trust in AFNs, limited research has been conducted to reveal nuances in the ways in which trust is reconvened and how a community gathering different types of AFNs is built amidst China’s sociocultural context. In particular, deeper understanding is needed in terms of the role of personal social connections in the community building behavior in AFNs.

Our research answers the question of how AFNs’ communities (i.e., customer base) are established and expanded in the Chinese social context. Through in-depth examination of six distinct cases, we found some important features in these communities. That is, they are composed of multiple networks of personal relationships, which we refer as *guanxi-circle*. The first half of the term, *guanxi*, is a prevalent socio-cultural construct in sociological, political, anthropological and business studies that describes personal interactions between two individuals. It refers to “an informal, particularistic personal connection between two individuals who are bounded by an implicit psychological contract to follow the social norm of *guanxi* such as maintaining a long-term relationship, mutual commitment, loyalty, and obligation” [36] (p. 306). Researchers have highlighted the utilitarian nature of *guanxi* and argued that it is based implicitly on mutual interests and benefits [37]. As Lee and Dawes [38] (p. 29) indicated, “*guanxi* lies at the heart of China’s social order, its economic structure, and its changing institutional landscape”. There are three types of *Guanxi*: family *guanxi* (equivalent to kinship), acquaintance *guanxi* (e.g., relationships with former classmates or colleagues), and strangers *guanxi* (relationships with people without common demographic attributes). Different forms of *guanxi* trigger different principles of interaction: altruism for family *guanxi*, reciprocity for acquaintance *guanxi* and exchange for strangers *guanxi* [39]. Trust is a major building block for *guanxi*, and *guanxi* also in turn facilitates the development of trust [40]. 

The second half of the term, circle, resembles the idea of social circle, but also differs from a social circle. *Guanxi-circle* is a group of people based on one kind of *guanxi*. Building on the utilitarian nature of *guanxi*, it applies to the reciprocity principle. *Guanxi-circle* can be divided into two types: the *guanxi-circle* based on common experience, and the *guanxi-circle* based on common interests. We use the term *guanxi-circle* as a conceptual tool to analyze the process of community building in AFNs. The concept not only has richer social cultural meanings than “social circle” in terms of the reciprocity principle and the associated one kind of relationship, but also implies an implicit linkage with the well-known term *guanxi*. As *guanxi* is fluid and changing, *guanxi-circle* also demonstrates a nonstatic feature. 

Many existing studies, with the exception of a few [28], exploring community building in AFNs have focused on the community at a macro level to examine how multiple AFNs form alliances to generate a transformative power to change the mainstream industrial food system [5,24]. By analyzing the use of social media among Chinese CSA farms, Chen and Tan [34] shows that social media plays a positive role in enhancing producer-member relationships and thus contributes to community solidarity. Martindale’s study demonstrates how Chinese AFNs convey specific material qualities of their food through social media to relieve the “trust pressure” they face [28]. Yet, these studies that inspired our research have not examined the micro level process of community building within these initiatives since their establishment and particularly the role of *guanxi* in this process. Building on the theorizations of trust and *guanxi*, the paper contributes to the literature by unveiling the nuances of AFNs’ successful strategies in community building. We argue that the community building of Chinese AFNs is a dynamic process that entails three distinctive stages, in which *guanxi* and trust are key building blocks. Through maneuvering the *guanxi* between the founder and their customers and among customers, these initiatives have managed to establish a reliable customer base.

## 3. Research Methods

### 3.1. Case Design 

We used an inductive and multiple-case research design. A case study is a preferred research strategy for studying complex social phenomena, particularly issues not well explained by existing theories [41]. In view of the fact that we knew little about the formation of new communities in AFNs, we attempted to explore this issue theoretically through case studies. Compared with single case study, multi-case studies can better verify or disprove inferences from other cases and thus provide a more robust and universal theory.

To understand how *guanxi-circle* shapes community, we employed a longitudinal design to track the community development process. This design required us to study community cases with rich historical archives and where people were willing to participate in multiple interviews, which were necessary to understand the temporal dynamics of community building behavior. Similarly, although the six cases in this study have been more successful than many other AFNs in China, they exhibit much variation in the ways in which their *guanxi-circle* emerged, such as failed actions and major mistakes, bringing useful variances to our theorization. 

We selected six AFNs cases for the following three reasons. (1) They cover the three major types of AFNs in China. AFNs were introduced to China in 2003 and began to win widespread acceptance in 2009 after the 2008 melamine milk scandal. In 2003, there were only three CSA farms, but the number grew to about 50 in 2009 and more than 2000 in 2022. Three of the major types of AFNs in China are CSA farms, farmers’ markets and social enterprises. Cases A and B are CSA farms. Cases C and D are farmers’ markets. Cases E and F are social enterprise; (2) They were all established around 2010, which is sufficiently early to allow longitudinal patterns to emerge and yet sufficiently recent to allow accurate, detailed data collection at the time of our study. (3) They are distributed in both southern China and northern China, which are geographically representative. Case A, C and E are located in North China Beijing, and Case B, D and F are from South China Guangdong Province. The founders include both men and women, considering the potential gender differences in community building behavior. The initiators of Case A, C, D and F are women, while Case B and F are men. Table 1 summarizes the diverse characteristics of these cases.

### 3.2. Data Collection 

In our data collection, we focused on tracking the community building behavior in all six cases during their first five years of establishment. We relied on two primary data sources: archives and interviews. We began data collection by gathering extensive archival data from both internal and external sources. The internal sources included all press releases since the establishment of the selected AFNs, internal reports and presentations, as well as archived videos and recordings made by executives on various occasions during those years. The external sources included media articles about each case found on *Baidu* (Baidu is a popular web search engine in China, similar to Google). Using their names of the AFNs as keywords, we collected 80 to 100 articles per case. If available, books and analytical reports about each case complemented these articles. Based on these very extensive archival data, we wrote up the history for each case.

Semi-structured interviews with internal and external informants constituted the second source of data. We conducted an average of 25 interviews per case. Each interview ranged from 30 min to 2 h in length. In total, 163 interviews were conducted from early 2014 to late 2021. The extensive interview data allowed us to identify critical stages in the formation of the *guanxi-circle*, and these stages were then cross-verified with those identified in the archival material. The selection of internal interviewees was based on three criteria: (1) they have been involved in the AFNs for a long time in their case, which enables them to provide a temporal perspective on the *guanxi-circle* formation; (2) they have been directly involved in at least one major *guanxi-circle* so that they could provide in-depth first-hand information, and (3) they represent different functions and hierarchical levels within the AFNs, which allows us to obtain diverse perspectives. In addition to these internal informants, we selected five types of external interviewees: former employees, volunteers, business partners, competitors (i.e., other CSA farms, farmers’ markets or food social enterprises who could provide information about these six cases), and industry experts. By interviewing diverse informants, the information was verified by several sources and thus mitigated the potential bias of any individual respondent.

### 3.3. Analytical Approach

We begin with an in-depth analysis of each case through the lens of our research question: How does *guanxi-circle* shape the evolution of the AFN’s community over time? We did not advance any hypotheses but read the cases independently to develop our own views by using tables and graphics. We then coded the interview data using NVivo to identify a list of key terms related to the community building behavioral process. After several group discussions, we synthesized the information and identified cognitive trust and emotional trust as the key tools of community enhancement. We then compared the insights with those from other cases to identify consistent patterns and themes. Data analyses led to a three-stage framework to explain how *guanxi-circle* helps to build the community of AFNs.

To build a grounded theoretical model, we followed Gioia et.al’s systematic approach to develop a theory that is empirically grounded [42]. This analytical approach had four distinctive stages of analysis. (1) *Open coding*: During the first stage, the interview transcripts were analyzed using line-by-line open coding with NVivo software, looking for evidence of informants’ perceptions and practices related to the community building effort. (2) *Developing general coding categories*: As the themes popped up frequently, to guide our data coding, we started to search the concepts, notions, and synonyms that better describe the links to the AFNs’ community building behavior in the Chinese context. (3) *Theoretical coding*: Theoretical codes conceptualized how the substantive codes may relate to each other as hypotheses to be integrated to explore which creative insights could best explain the data and we identified 10 theoretical themes out of the categories. These themes included “ecological values”, “social responsibility”, “personal relationships”, “life experiences”, “sustainable farming”, “fair trade”, “risk sharing”, “*guanxi*”, “cognitive trust”, and “emotional trust”. (4) *Axial coding*: At this stage, we reanalyzed the interview transcripts, archive data, and secondary data with a revised focus on these theoretical themes. We also moved iteratively between the literature and data for multiple rounds to come up with the aggregated theoretical dimensions. Eventually, we identified *guanxi-circle* as the overarching umbrella that explains the diverse mechanisms of community building practices. The aggregated *guanxi-circle* themes included cognitive trust and emotional trust that empowered the AFNs operators to build, expand and maintain their communities. 

We also used a social network analysis (SNA) method to further unpack the AFNs communities by analyzing their core mechanisms and key turning points. The SNA method was originally proposed to measure the structure of networks; Granovetter further developed this method to analyze the informal networks and weak ties [43]. In our study, we build a *guanxi* matrix to reveal the progress of building secondary *guanxi-circle* that eventually constituted the AFN community. In the SNA method, it is marked as 0 if there is no *guanxi* between two actors, showing no edge between the two nodes. Otherwise, it is marked as 1. In this way, we built a 0–1 *guanxi* matrix which revealed the structure of the *guanxi* network. Through network topology analysis, a graph could be constructed to show the *guanxi* connecting community members clearly at the micro level.

## 4. Findings

Through careful examination of these cases, we conclude that the formation of communities in Chinese AFNs has typically gone through three stages: (1) set up of the initial *guanxi-circle* based on the cognitive understanding of food safety risks and the emotional empathy with the founder of the alternative food initiative; (2) expansion of the initial *guanxi-circle* through the fostering of *guanxi*, which is designed to improve members’ cognitive trust and emotional trust, and attract new members to join the community during this process, and (3) formation of a secondary *guanxi-circle* promoted by key members who fill the structural holes. Structural hole refers to the gap between two individuals with complementary resources or information in the social network. They are the “gaps” in the network structure. The person who occupies the structure hole (i.e., fills in the gap) will be able to bridge the two individuals [44] (Burt, R.S., p. 659–663). These processes eventually lead to a constructed community around a certain initiative. In the next section, we unveil how these three stages unfold in specific cases.

### 4.1. Building the Initial Guanxi-circle

In China’s emerging ecological food market, founders of AFNs must carve out a coherent cognitive space to secure an original customer base. Developing cognitive trust involves a step called “cognitive leap”, which represents the point at which people do not need any additional evidence or rational explanation to form the trust in a particular object [32,45]. Therefore, the strategy of AFNs to gain customers’ cognitive trust has been to provide them with additional rational explanation. However, AFNs are a new and unfamiliar concept for most Chinese consumers, which often leads to confusion. In response to this situation, the founders of AFNs often use self-explanatory value-loaded language to convey to potential customers their environmental and social responsibilities. In many occasions, this often turns out to be a storytelling strategy, where AFNs founders would deploy their personal *guanxi* in an attempt and strive to gain both cognitive trust and emotional trust from potential customers.

We find that in these cases, the ecological values and social responsibility associated with AFNs operations are crucial keys to build cognitive trust among potential customers. The founders’ ecological values, which guarantee food safety, are demonstrated through their various ecological farming techniques. For example, they encourage farmers not to use synthetic fertilizers and chemical pesticides; they also emphasize inter-cropping, and the seasonality of fruits and vegetables, and use other means to protect biodiversity. The ecological approaches are especially important given the widespread public concern over food safety in China, particularly over chemical residues in food that has been conventionally produced. Chen highlights that the desire to live a healthy life through the consumption of safe food is an important factor in attracting consumers into the community [13]. The following quotes demonstrated this finding.

*“You know, unsafe food is caused by the abuse of chemical fertilizers, pesticides and hormones. This farm promises not to use those things. It makes me feel that food safety is guaranteed. Fortunately, I find a place for my children to buy safe food!”*

(Consumer Ms. Liu from Case A in 2017)

*“This farmers’ market is special, different from regular food markets. The vendors are all from suburbs of Shenzhen. They sell seasonal food grown by local farmers, healthy and environmentally friendly. I wrote several articles to recommend them to my readers.”*

(Health expert Mr. Yu from Case D in 2018)

The founders of AFNs are also aware of consumers’ interest in food safety and intentionally use it to build cognitive trust.

*“In the context of food safety crisis, we should emphasize the safety of food through AFNs. However, AFNs have many goals, such as sustainability, localism, support for rural development, etc. But safe food is the aspect most likely to attract consumers and win trust at present.”*

(Founder from Case F in 2017)

Social responsibility is demonstrated through an emphasis on risk sharing, fair trade, and conscientious (*liangxin*) production. Conscience (*liangxin*) in the Chinese context means a sense of right and wrong in one’s heart; a sense of responsibility for good deeds and often thought to arouse guilt and regret for bad deeds. Operators embed these values into their interactions with customers, for example through marketing activities. The values are well received by some customers.

*“As a CSA farm, we promise consumers that we produce in a liangxin way and ensure the safety of our food. Consumers share the risks with us through prepayment, which will enable us not worry about natural risks and market risks. We will do a good job, produce safe food and let time test our promises.”*

(Founder from Case B in 2018)

Emotional trust is established through interpersonal trust and the connections with customers’ memorable personal life experiences. Interpersonal trust usually builds on previously accumulated trust that people already feel toward the founder or team member and through multiple face-to-face interactions between operators and consumers. People’s memorable experiences are used in the AFNs’ promotional content activities, which highlights how products are relevant to customers’ daily life. This connection with customers’ life experience is often achieved through various publicity slogans, such as *“Melon left, beans right, cultivated under the green leaves”, “Your safe food is what we guarantee you”, and “The taste of childhood”*. These and other similar expressions represent more than advertising slogans. Compared to excessive conventional marketing commercials that encourage impulse purchases, these expressions are laden with sentiments that bring community members closer. They are carefully phrased to create a welcoming connotation, making the relatively exotic alien concept of AFNs familiar. Social media, such as Microblogs and WeChat, are often deployed to convey these messages. Despite decades of urbanization in China, most urban settlers are migrants from rural areas and many of them feel nostalgic about the countryside life, or a strong desire to reconnect with the rural through food. The discourses put forth by AFNs help to awaken the idealized rosy childhood memories of food and build emotional connections.

*“Professor He and I have been friends for many years. Although I don’t know what AFNs are, I don’t think she could do anything wrong. Six years ago (in 2013), she invited me to participate. I agreed without hesitation. Well, friends should help each other.”*

(Consumer Mr. Zhou from Case E in 2019)

Throughout the trust building process, members have developed a gradual understanding of AFNs. Through pesticide-free agriculture, integrated crop and livestock farming, pre-pay schemes, and fair trade, AFN founders have created a rational cognitive benchmark within the initial *guanxi-circle*. Past and newly created *guanxi* between founders and consumers, and the awakening experiential memories, help them deepen the emotional trust of members in the initial *guanxi-circle*. Hence, the cognitive trust people develop in the safe food produced and channelled through AFNs and the emotional trust in the founders, constitute the basis for the formation of initial *guanxi-circle*. 

### 4.2. Strengthening and Expanding the Initial Guanxi-circle

The second step in community building of AFNs is the strengthening and expanding of the initial *guanxi-circle* through the fostering of the *guanxi* cultivation, which is designed to increase the frequency of interactions, deepen the mutual understanding and ultimately enhance trust. Developing cognitive trust and emotional trust can cultivate the *guanxi*. In our case, we found that cognitive trust is enhanced through the fostering of the *guanxi* cultivation which could be conducted through frequently posting on social media such as WeChat and Microblogs, interacting intensively with their customers, addressing crisis in public relations and pursuing third-party endorsement. To gain emotional trust, the *guanxi* can be cultivated through the organization of summer camp activities, salons and dinner gatherings, the preparation of refined information cards and the meticulous customer service. *Guanxi* cultivation plays an important role in the expansion of initial *guanxi-circle*, determining whether existing consumers can be retained and whether existing consumers are willing to introduce new members to the AFNs. As founders have different levels of resources endowments and personalities, they cultivate the *guanxi* in different ways.

The founders in all six cases used social media, mainly WeChat and Microblogs, to nourish consumer’s trust in the original *guanxi-circle*. In cases A and B, they frequently update social media contents, focusing on the germination, blossoming and ripening fruits of the crops and enhancing customers’ awareness of the changing seasonality on the farm. The founders of the farmer’s market (cases C and D) always post articles and photos about farmers’ farming and seed breeding activities, and organize farm visits. The content of the social media posts for social enterprise cases (cases E and F) mainly relates to farms and cooperatives with which they collaborate.

*“On WeChat, she often provides updates about what’s happening on the farm, and her updates sometimes fully take over my screen. For example, pig barns and the blue flowers bloom…the children do not want to leave the farm as they are addicted to playing in the mud. We always upvote each other’s WeChat posts. Haha.”*

(Consumer Ms. Wang from Case A in 2019)

Original articles published on WeChat and Microblogs that demonstrate producers’ professionalism and artisan spirit enhance customers’ trust.

*“I learned about this honey from one article published on their WeChat account. The introduction is very detailed. I was deeply moved by their narratives. They were not urging you to buy but just quietly showcased how they were caring for the land and food cautiously and piously. I came to first realized that the Chinese bumblebee is so important.”*

(Consumer Ms. Liu from Case E in 2020)

Occasional incidents of trust crisis can also be transformed into great opportunities to raise cognitive awareness among customers. The two founders of Case A, for example, responded well to a crisis they faced through which they reinforced the solidarity among members of their initial *guanxi-circle*. In this case, one farmer used chemical pesticides secretly in order to get better yield, but chemical pesticides had always been prohibited on the farm. After the founder learned that all the vegetables were sprayed, she pulled out and destroyed all of them. The handling of this incident won wide support and stronger trust of many customers, as the following quotes show.

*“At that time (in 2012), Ms. Shi rushed into the fields angrily and cleared out all the [sprayed] vegetables. But not enough vegetables were left for the delivery in the following week…However, after consumers knew this thing, they were very happy because she kept her promise. And they trusted her even more… thank God she did that.”*

(Hired Farmer Mr. Ma from Case A in 2018)

*“It was wise for her to have done that, which demonstrated her adherence to principles. At that time, AFNs were still new and everyone would be sceptical. That incident cleared people’s doubts and they trusted her more.”*

(Competitor Mr. Wang who worked in a Beijing’s Agricultural company in 2018)

As a result, more and more consumers joined Case A’s initial *guanxi-circle* which was built upon the teacher-student *guanxi*. Many of the initial members were Ms. Shi’s teachers and classmates at the university where she graduated. The initial *guanxi-circle* of Case A has been enhanced and expanded.

In terms of third-party endorsements, Case B acquired organic certification through the Organic and Food Development Center based in Nanjing, the most reputable organic certification agency in China. The other five cases claimed that they grew their food organically but did not have organic certification. Although many small ecological farms in China do not pursue organic certification, we found that third-party endorsements did give some consumers more confidence over the quality of food.

*“I know that they (Case B) has organic certification. Many CSA farms are producing organically. But nobody knows whether they have met organic standards. I also have my own restaurant and have made stable purchase here for two years. The organic certification matters much as my food purchase requires a full certificate.”*

(Business partner & Consumer Mr. Xia from Case B in 2021)

Organizing on-farm activities is another commonly used strategy to strengthen the initial *guanxi-circle*. Some popular consumer activities include “One-meter Garden”, “Festival DIY” and “Farm Summer Camp”. During these interactive processes, the farm managed to deepen the interpersonal bonds between the farm and their customers. The community was also strengthened as many customers also made new friends during these events.

*“The summer camp activities were good. Children were very excited to see lambs and puppies. They also learned about the leaves of okra, pepper and tomato. The mosquitoes were a little bit annoying, but the children were very happy.”*

(Consumer Ms. Liang from Case A in 2020)

*“I’m a nutritionist. There was a moon cake event during the Mid-Autumn Festival. I took the child there and met another nutritionist mother. We discussed a lot on skills and tips for preparing food for children with safe ingredients.”*

(Consumer Ms. Shang from Case B in 2019)

Case C holds a “salon sharing” (seminar) once a week, covering topics such as eco-agriculture, food safety, social development and NGO operation. They have invited producers, scholars, and consumers from Europe, USA, Japan, and Africa to speak at these events. This regular salon offers opportunities for face-to-face communication. More and more audiences have become consumers and some even became staff members. In this way, the initial *guanxi-circle* has been expanded.

*“I think the salon is very good. Many producers also come to share [their stories]. I really like Mr. Leho and Mr. Rice. They are both cool producers.”*

(Consumer Ms. Li from Case C in 2020)

Case E has expanded its *guanxi-circle* by organizing public events, such as conferences, workshops and trainings. Both Case E and Case F earned customers’ support through intimate, meticulous and ingenious customer service, as demonstrated by the following quotes.

*“My wife was deeply moved by professor He’s story (Note: Professor He Huili was a deputy county governor in Lankao County, Henan Province who tried to organize farmers to grow rice with reduced usage of chemicals and brought the rice to Beijing for sale in 2005. The story was covered by some influential newspapers as “Professor Selling Rice”.). She introduced her friends to support ecological products.”*

(Consumer Mr. Hua from Case E in 2018)

*“In the millet package I received, there was a thank card that was handwritten by the farmer, very impressive and meticulous.”*

(Consumer Ms. Meng from Case F in 2019)

These practices show that *guanxi* cultivation can be approached from building both cognition trust and emotion trust. The founders picked the most effective approaches of *guanxi* cultivation based on their own advantages. For example, the founder of Case A is a well-known figure in China’s ecological farming sector, so her reputation was often referenced to maintain frequent communications with initial *guanxi-circle* through social media. Case B used their high level of agricultural expertise to gain appreciation, and endorsed itself through third-party certification. Case A and Case F both responded swiftly and effectively to public relations crises. Their sincere attitude and adherence to the ecological principles of AFNs transformed the crises into trust-building opportunities. As *guanxi* cultivation advances, the initial *guanxi-circle* is gradually strengthened and expanded.

### 4.3. Building a Secondary Guanxi-circle

The second stage constitutes the process of strengthening the trust of members from the initial *guanxi-circle*, while the third stage involves a process in which these key members of that initial *guanxi-circle* expand their *guanxi* network and bring in new members to form a secondary *guanxi-circle*. A key element in making the transition from stage two to stage three is the changing role of key members from customers to conveners of the new *guanxi-circle*. While start-up members are at the center of the initial *guanxi-circle*, it is the key members of the initial *guanxi-circle* who are at the center of the secondary *guanxi-circle*. The third stage cannot happen without the *guanxi* cultivation of the second stage that prepared the key members (enhanced trust, shared visions and solid knowledge) for expanding their networks. The number of community members usually increases significantly from less than 100 in the second stage to more than 600 in the third stage.

To uncover the dynamic formation and evolution process of the community building in AFNs, the SNA method was employed to analyze relatively enclosed and complete *guanxi* data. As Case A has rich *guanxi* data between customers, we use it to demonstrate the third stage of community building. Case A has more than 600 customers, and they can be divided into 11 *guanxi-circle*. The 11 groups of *guanxi-circle* correspond to 11 WeChat groups that function as buying clubs and food delivery stations. WeChat groups provide a virtual space for these reconstructed guanxi-circle built around key members and an instrument to enhance the community. We found that people living close have much higher chance to be in one *guanxi-circle* as spatial proximity implies higher chance of having children studying in the same school or being the member of the same community library or gym. Despite this, the *guanxi-circles* are not completely overlapping with the pick-up groups. Based on the availability of interviewees and the completeness of the data, we interviewed 60 members to build a *guanxi* matrix (Table 2). Zero indicates that there is no *guanxi* between two actors, showing no edge between two nodes; otherwise, it is marked as 1. The two parties must be acquaintances in order to be given a value of 1 in the matrix. 

However, given that it is difficult to quantify the extent to which people know each other well, the *guanxi* reflected in the matrix is based on self-reported information, and informants must confirm that “they know each other well” in order to be considered to have a *guanxi*. Regarding the sample size (60 in this case), Eisenhardt believes that it depends on the degree of theoretical saturation [41]. It is necessary to increase the number of participants in the samples until theoretical saturation is reached. In our case, the sample size reached the degree of theoretical saturation, as the connections between participants clearly revealed the first and second stages in which the *guanxi-circle* was completed.

**Figure 1:**
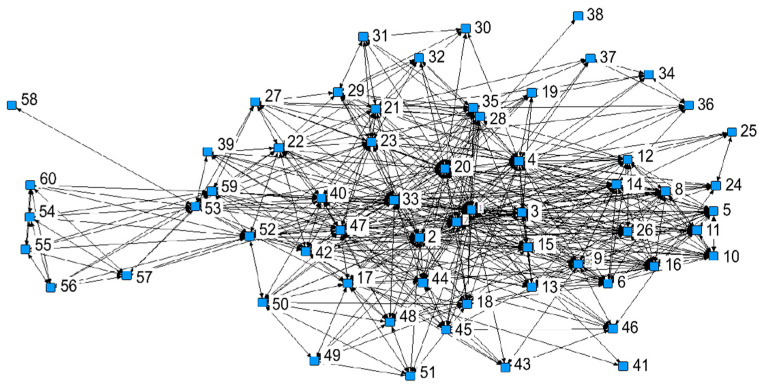
The map of *guanxi* matrix in Case A.

Figure 1 shows the network of *guanxi* among shareholders in Case A. We can see that member 1 occupies the center of the network, and members 1, 3, 4, 7, 47 and 53 are key actors, with dense connections around them. This pattern reflects their central roles in community building. Member 1 is the founder. Members 3 and 4 are responsible for marketing, and member 7 is the sales director. These three actors are close to consumers, so many consumers joined the network (*guanxi-circle*) through them. Member 47 works at the local agricultural bureau, and the farm needs his help for handling administrative procedures such as subsidies. Member 53 is a promoter for a farmers’ market, and she is Christian; therefore, she plays a central role in building a Christian fellowship *guanxi-circle* within the CSA community.

In addition to these few obvious key actors, members 2, 20, 22, 42, 52 and 59 occupy the structural holes in the social network. For example, member 2 is member 1’s husband, and owns a company that offers IT service for CSA farms, so he occupies a different *guanxi-circle* beyond the CSA community, and thus he can deploy his connections to set up new *guanxi-circles*. These members are always motivated to share the safe food produced through AFNs with their social networks for reciprocal benefits. For example, member 20 introduced the farm to his family and friends to show them how to access safe food in today’s risky food environment. 

The factional analysis of the actors in the community clearly reveals the existence of multiple *guanxi-circles*, the positions and roles of different members in each *guanxi-circle* and the *guanxi* that exists between these *guanxi-circles*. Table 3 shows the results in which four subgroups are identified, indicating four *guanxi-circles*.

The first faction contains 19 actors, who are all founding team members, except members 20 and 26. As previously explained, member 20 contributed greatly to the formation of the initial *guanxi-circle* and later on the promotion of secondary *guanxi-circle*. Member 26 is member 3’s fiancée, and she plays an important role in promotion. This *guanxi-circle* has contributed greatly to the community building. We name this *guanxi-circle* as a “start-up team” *guanxi-circle*. 

The second faction includes 14 actors. Members 17 and 19 are middle-class customers who pursue a healthy life. Member 24 is a dietician, and member 25 is her friend; both are deeply concerned about health and nutrition. Members 33, 39, 40, and 42 are NGO personnel who advocate ecological agriculture. Member 43 to 46 are elementary school leaders who run a food education program in cooperation with the farm. Member 47 is a government official committed to eco-agriculture. Therefore, we name the second distribution “healthy living” *guanxi-circle*. 

The third faction contains 11 actors. All the members are Christian consumers, who question the standardization of agriculture and pay attention to the natural attributes of food. These people have significant beliefs, so we name that group as the “belief” *guanxi-circle*. 

The fourth faction contains 13 actors. Most of them belong to families with young children. Member 27 is member 22’s wife. Members 28 to 32, 34 to 37 are friends and teachers of the founder. They are sensitive to food safety, care for children, and are well-to-do. Most of these people choose to join the community for the health and education of their children. We name it as “young parents” *guanxi-circle*. 

The analysis above shows that the community of a CSA is composed of many *guanxi-circles* that are constructed around different kinds of social connections. The potential reciprocal benefits motivate key members to share CSA products, information or resources with people in their social circles. Key members also occupy the structural holes that exist across different *guanxi-circles*, which in turn allows them to interweave, strengthen and expand their connections within the community. 

## 5. Conclusions

Community building behavior is critical for establishing the trust between farmers and consumers in AFNs. Existing studies on community building behavior in AFNs focused on a macro level community organizing across multiple AFNs that forms a transformative alliance to change the industrial food system. However, few studies examined the community building behavior within specific AFNs and the role of the traditional social-cultural construct, *guanxi*, in the process. This paper fills this gap by interrogating the community building behavior in six AFNs cases in China. 

**Figure 2:**
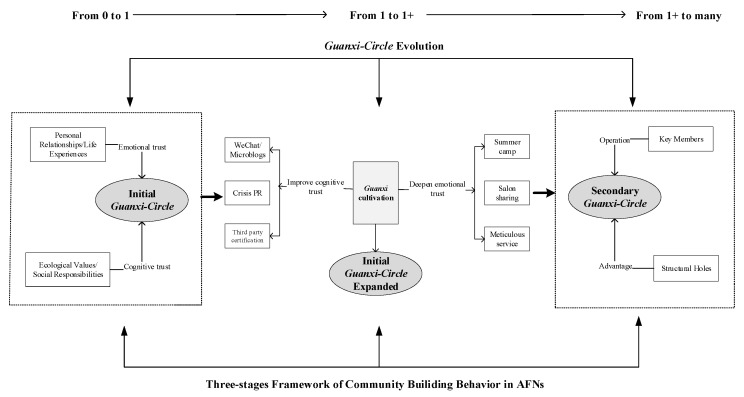
Three-stages framework of community building in AFNs.

Building on the sociological construct of *guanxi* in the Chinese context, this paper proposes the concept of *guanxi-circle* to denote the cluster of community members who share with a specific kind of social relation. Our surveys among Chinese AFNs communities identify several types of *guanxi-circles*, such as a group of alumni (schoolfellow *guanxi-circle*), or a group of people sharing the same religion groups (Christian/Buddhist *guanxi-circle*), and a group of people pursuing a healthy and sustainable lifestyle (LOHAS *guanxi-circle*). We conclude that the community building of Chinese AFNs has typically experienced three stages, including the establishment of initial *guanxi-circle*, the expansion of this initial *guanxi-circle* and the development of multiple secondary *guanxi-circles*. The formation of new *guanxi-circles* in both the first and the third stage depends on the enhancement and restructuring of pre-existing *guanxi*. The expansion of an AFN community can occur on the basis of existing relationships, particularly relationships of trust, among members. The pre-existing *guanxi* greatly enhances the efficiency of AFNs’ promotional activities and also provides crucial leverage for retaining customers. Finally, all the *guanxi-circles* together and the interactions among them constitute the community of AFNs, as Figure 2 shows. 

Our case studies also demonstrate that the community building in AFNs is more than a simple accumulation of *guanxi-circles* or marketing practices. It is, indeed, the process of converting and restructuring a pre-existing *guanxi* into a community. Cultivating cognitive trust and emotional trust is crucial in determining the conversion process. In addition, our research unveiled the dynamic interactions that take place beyond commodity exchange between founders of these AFNs and their members. The interactions between members from different *guanxi-circles* bring them together, providing them with opportunities to cultivate new *guanxi*. The dynamics between founders and members and among members themselves allow the AFNs to construct a sense of solidarity formed around common interests and goals.

By unpacking the success of Chinese AFNs, this research contributes to food and trust studies by demonstrating the central place of *guanxi* in scaling up ecological food initiatives. It calls for more attention to the social embeddedness of these initiatives [35]. This social embeddedness is not only exemplified by how broad social changes such as urbanization, rural restructuring and food safety crisis shape the emergence and development of alternative food initiatives, as previous studies have demonstrated, but it also reflects how essential *guanxi* is for the success of these initiatives and its critical role in shaping how the community is built and maintained.

AFNs provide a potential path for transitioning towards a more sustainable food system. On the one hand, AFNs use a sustainable production method which constructs a collaborative relationship between humans, nature and the land, promoting mutual assistance and cooperation between distinctive groups. On the other hand, AFNs creates a viable path for urban agriculture. In the modern “capitalized” agricultural system, urban and rural areas are not only segregated spatially, but food production and consumption are also divided psychologically and culturally, which has bred mutual discrimination and distrust. Through AFNs, producers with strong social and environmental values and entrepreneurial spirit could involve the urban community in sustainable and safe food production. Meanwhile, cities could incentivize meansingful urban-rural interactions, mutual assistance, and mutual trust by providing services such as technical guidance, educational activities, and leisure and entertainment opportunities. Their potential in promoting the integrated development of urban and rural areas over time should not be underestimated.

Our research on the community building strategies of these successful communities from a stakeholder perspective generates two recommendations for the scaling up of AFNs in the Chinese context. First, the founding and executive team should explore their existing social connections and cultivate them as “seeds” for growing their initial customer base. Second, AFNs practitioners should tap into and mobilize “key members” in the initial *guanxi-circle*. Key members occupy structural holes, and thus have power to develop secondary *guanxi-circles* by linking their different social networks. The key for establishing a secondary *guanxi-circle* lies in whether key members can be discovered and mobilized. In this sense, the process certainly opens up more space for these founders to take initiatives and play a more proactive role in the development of AFNs.

Future research on community building behavior could examine how the breakdown of a *guanxi-circle* affects community development and how individuals in an initial *guanxi-circle* could be motivated to become key members. It would also be useful to conduct large sample survey to test the findings of this study.

## Figures and Tables

**Table 1:** Description of Cases.

	Case A	Case B	Case C	Case D	Case E	Case F
**Types**	CSA Farm	CSA Farm	Farmers’ Market	Farmers’ Market	Social Enterprise	Social Enterprise
**Founder**	Female, PhD, opinion leader, Socialist, studied abroad	Male, entrepreneur, engaged in finance and real estate, 10 years of experience in organic agriculture	Female, Journalist, Opinion Leader	Female, entrepreneur, Christians leader with strong integrity and concern for public interest	Organized by Professor He, cares about food safety and rural development	Male, entrepreneur, agriculture science expert, has rich CSA experience
**Location**	Beijing North China	Huizhou South China	Beijing North China	Shenzhen South China	Beijing North China	Guangzhou South China
**Team Numbers**	21	40	5	6	18	30
**Sources of start-up capital**	Self-raised +10 shareholders	108 shareholders	———	Sponsors and four sponsoring agencies	Institutional funding	Self-raised
**Operating conditions**	Established in 2012, 2014 in balance, annual turnover 5 million Chinese Yuan, 600 consumers	Established in 2013, has organic certification, 2000 consumers, rapid development	Founded in 2010, held twice a week, 40 cooperative farms and enterprises	Established in 2012, opens once a month, 20 cooperative farms	Established in 2006, organized loosely, slow development, 7 cooperatives farmers	Established in 2008, 20 farming partners, more than 5000 customers, turnover of 6 million Chinese Yuan

**Table 2:** 60 × 60 *guanxi* matrix in Case A.

	Num.1	Num.2	Num.3	Num.4	Num.5	…	Num.59	Num.60
Num.1	0	1	1	1	1	…	1	0
Num.2	1	0	1	1	1	…	1	0
Num.3	1	1	0	1	1	…	1	0
Num.4	1	1	1	0	1	…	0	0
Num.5	1	1	1	1	0	…	0	0
…	…	…	…	…	…	…	…	…
Num.59	1	1	1	0	0	…	1	0
Num.60	0	0	0	0	0	…	1	0

Note: members are not fully listed due to the large size of the table.

**Table 3:** Factional analysis of *Guanxi-circle*.

Number of Factions	Members in Each Faction
1	1–16, 18, 20, 26
2	17, 19, 24, 25, 33, 39–47
3	38, 41, 52–60
4	21–23, 27–37

## Data Availability

The data of this study are available from the corresponding author upon reasonable request.
